# Adherence to the objectives of the Safe Surgery Saves Lives Initiative: perspective of nurses^*^

**DOI:** 10.1590/1518-8345.2711.3108

**Published:** 2019-01-31

**Authors:** Larissa de Siqueira Gutierres, José Luís Guedes dos Santos, Sayonara de Fátima Faria Barbosa, Ana Rosete Camargo Maia, Cintia Koerich, Natalia Gonçalves

**Affiliations:** 1 Hospital Baia Sul, Centro Cirúrgico, Florianópolis, SC, Brazil.; 2 Universidade Federal de Santa Catarina, Departamento de Enfermagem, Florianópolis, SC, Brazil.

**Keywords:** Patient Safety, Surgicenters, Operating Room Nursing, Quality of Health Care, Practice Management, Health Management, Segurança do Paciente, Centros Cirúrgicos, Enfermagem de Centro Cirúrgico, Qualidade da Assistência à Saúde, Gerenciamento da Prática Profissional, Gestão em Saúde, Seguridad del Paciente, Centros Quirúrgicos, Enfermería de Quirófano, Calidad de la Atención de Salud, Gestión de la Práctica Profesional, Gestión en Salud

## Abstract

**Objective:**

to measure the adherence to the objectives of the Safe Surgery Saves Lives Initiative in surgical centers from the perspective of nurses.

**Method:**

cross-sectional study, developed through an online survey via the Google Forms^®^ platform. The study participants were 220 nurses from surgical centers in different regions of Brazil. The data were collected through a socio-professional characterization form and a questionnaire in which the participants indicated their level of agreement in relation to the fulfillment of the objectives of the Safe Surgery Saves Lives Initiative. Data analysis was performed using descriptive statistics.

**Results:**

objective 1, The team will operate on the correct patient at the correct site, presented the highest levels of total agreement (n = 144; 65.5%) and partial agreement (n = 52; 23.6%). Objective 10, Hospitals and the public health systems will establish routine surveillance of surgical capacity, volume and results, obtained the lowest percentages of total (n = 69, 31.4%) and partial agreement (n = 81, 36.8%).

**Conclusion:**

adherence to the objectives of the Initiative is adequate, but there are weaknesses, especially in relation to the prevention of never events.

## RESUMO

**Objetivo:**

mensurar a adesão aos objetivos do Programa Cirurgias Seguras Salvam Vidas em centros cirúrgicos a partir da perspectiva de enfermeiros.

**Método:**

estudo transversal, desenvolvido por meio de um *survey on-line* via plataforma *Google Forms* ^®^. Os participantes da pesquisa foram 220 enfermeiros de centros cirúrgicos de diferentes regiões do Brasil. Os dados foram coletados por meio de uma ficha de caracterização socioprofissional e questionário em que os participantes indicavam seu nível de concordância em relação ao cumprimento dos objetivos do Programa Cirurgias Seguras Salvam Vidas. A análise dos dados foi realizada por meio de estatística descritiva.

**Resultados:**

o objetivo 1, operar o paciente certo e local cirúrgico certo, apresentou os maiores níveis de concordância total (n=144; 65,5%) e parcial (n=52; 23,6%). O objetivo 10, o hospital e os sistemas de saúde pública estabelecem vigilância de rotina sobre capacidade, volume e resultados cirúrgicos, obteve os menores percentuais de concordância total (n=69; 31,4%) e parcial (n=81; 36,8%).

**Conclusão:**

a adesão aos objetivos do Programa é adequada, mas há fragilidades especialmente em relação à prevenção de *never events*.

## RESUMEN

**Objetivo:**

medir la adherencia a los objetivos del Programa Cirugías Seguras Salvan Vidas en centros quirúrgicos desde la perspectiva de enfermeros.

**Método:**

estudio transversal, desarrollado por medio de *survey on-line* vía plataforma *Google Forms* ^®^. Los participantes de la investigación fueron 220 enfermeros de centros quirúrgicos de diferentes regiones de Brasil. Los datos fueron recolectados por medio de una ficha de caracterización socioprofesional y cuestionario en que los participantes indicaban su nivel de concordancia en relación al cumplimiento de los objetivos del Programa Cirugías Seguras Salvan Vidas. El análisis de los datos fue realizado por medio de estadística descriptiva.

**Resultados:**

el objetivo 1, operar el paciente correcto y local quirúrgico cierto, presentó los mayores niveles de concordancia total (n=144, 65,5%) y parcial (n=52; 23,6%). El objetivo 10, el hospital y los sistemas de salud pública establecen vigilancia de rutina sobre capacidad, volumen y resultados quirúrgicos, obtuvo los menores porcentuales de concordancia total (n=69; 31,4%) y parcial (n=81; 36, 8%).

**Conclusión:**

la adherencia a los objetivos del Programa es adecuada, pero hay fragilidades especialmente en relación a la prevención de *never events*.

## Introduction

Surgical centers are considered to be complex and high-risk units, susceptible to errors and adverse events that can lead to death or complications for patients. In developed countries, the rate of major complications in surgical procedures is 3-16% and the mortality rate is 0.4-0.8%; approximately half of these events may be considered preventable. In developing countries, mortality rates of 5 to 10% are estimated in large surgeries^1^.

In view of this scenario, in 2009, the World Health Organization (WHO) issued guidelines for the implementation of a universal protocol for the safety of surgical patients. The guideline was developed after the Safe Surgery Saves Lives Initiative and was translated into Brazilian Portuguese by the National Sanitary Surveillance Agency (ANVISA) and released in 2010^1^ ^-^ ^2^.

From this global initiative, the theme has gained broad projection through television media and social networks, widening the debate between specialists, health professionals and patients. The Safe Surgery Saves Lives Initiative^1^ aims at reducing the number of deaths and surgical complications and contemplates 10 essential goals to guarantee patient safety. This set of objectives can be considered as a tool for safety in the practice of health professionals, assisting them in the development of actions aimed at reducing errors in care processes^1^ ^,^ ^3^.

The implementation of a patient safety program in a health institution goes beyond the application of questionnaires and achievement of targets. Culture should be included in the mission and values of the health institution and leaders should understand the practice of patient safety as an indicator of quality of care. In this context, nurses are better able to identify the risks to which patients are exposed in the surgical center and, therefore, lead to the incorporation of a safe surgery culture and adherence to the objectives of the Initiative^3^ ^-^ ^5^.

National and international researchers have highlighted the need for research on how to improve the organizational culture of patient safety and to evaluate the evolution of the implementation of processes for improving surgical care^2^ ^-^ ^6^. However, according to a recent review of publications related to patient safety in the hospital environment, only 3.5% of the studies approached the subject of safe surgery, especially with regard to adherence and/or patient safety culture among professionals^6^. Thus, there is the need to deepen the knowledge about the adherence of health professionals to the Safe Surgery Saves Lives Initiative.

Adherence can be defined as the adoption and maintenance of good practices for quality and patient safety in health services, which requires from the professional technical knowledge, ethical attitude and motivation^7^. Thus, considering that surgical center nurses in Brazil are the managers of this sector and have a fundamental position in developing strategies for the safety of the surgical patient, the outlined research question is: How is the adherence of health professionals to the objectives of the Safe Surgery Saves Lives Initiative from the perspective of surgical center nurses?

The objective of the present study was to measure adherence to the objectives of the Safe Surgery Saves Lives Initiative in surgical centers from the perspective of nurses.

## Method

This is a cross-sectional study developed through an online survey for surgical center nurses from different regions of Brazil.

Data collection was performed from June to August 2017 via the Google Forms^®^ platform. The choice of a virtual questionnaire had the objective of maximizing the data collection, since Internet surveys represent an economical alternative that makes it possible to overcome geographical barriers and increase the number of study participants^8^.

For composing the research sample, the link with the questionnaire was sent by e-mail to registered nurses at the Brazilian Society of Surgical Center, Material and Sterilization Center and Post-Anesthetia Recovery (SOBECC in Portuguese) and at the Brazilian Nursing and Patient Safety Network (REBRAENSP in Portuguese). The sending of this e-mail was made directly by the aforementioned entities, and it is not possible to specify the total number of participants enrolled in this stage of the research. 

In a complementary manner, the study’s main researcher sent 341 e-mails with the questionnaire link to participants of the Brazilian Hospital Network with Patient Safety Center (NSP in Portuguese) registered with the Brazilian Agency of Sanitary Surveillance (ANVISA in Portuguese). The Regional Nursing Councils (CORENs) and the state sections of the Brazilian Nursing Association (ABEN) were also requested to send the questionnaire link to their members. These institutions were chosen for bringing together potential study participants.

In order to broaden access to research and, therefore, include non-registered nurses in the aforementioned institutions, the research link was also shared for WhatsApp^®^ groups and contacts to which the researchers had access and who were related to work in health/surgical centers. In total, 205 messages were sent via WhatsApp^®^. The link was also shared on social networks, such as Facebook^®^, LinkedIn^®^ and Instagram^®^, reaching more than 23 thousand people, of whom 219 clicked on the link.

Based on these strategies, we sought to include the largest number of nurses working in surgical centers in Brazil. In view of the absence of previous literature to estimate the number of nurses working in surgical centers at a national level and since the questionnaire was not restricted to the mailing lists, it was not possible to estimate a sample calculation. Thus, we obtained a non-probabilistic convenience sample composed of 248 nurses who answered the questionnaire.

We included nurses with at least three months of professional experience in surgical center and who were working in this sector at the time of the study. These inclusion criteria were informed to the participants at the time of the invitation to respond to the online questionnaire. Questionnaires with incomplete and duplicate information were excluded, that is, when the same participant answered the questionnaire more than once. Duplication of answers was assessed by auditing participants’ e-mail records, and the last response received was included.

The data collection instrument was composed of two parts, namely: a characterization form with variables about the socio-professional profile of the nurses (gender, age, experience in surgical center, training, country region, type of institution where they worked, weekly workload, type of professional performance and information about the work, such as the number of surgical rooms under the nurse’s responsibility and number of surgeries).

In the second part, a questionnaire was drawn up in which participants indicated their level of agreement regarding the fulfillment of each of the 10 objectives of the Safe Surgery Saves Lives Initiative in their current workplace. For the response, a Likert type scale was used with five response options: I Totally Disagree (TD), I Partially Disagree (PD), Neutral (N), I Partially Agree (PA) and I Totally Agree (TA). The 10 goals of the Safe Surgery Saves Lives Initiative^1^ are: (1) The team will operate on the correct patient the correct site; (2) The team will use methods known to prevent harm from administration and anesthetics, while protecting the patient from pain; (3) The team will recognize and effectively prepare for life-threatening loss of airway or respiratory function; (4) The team will recognize and effectively prepare for risk of high blood loss; (5) The team will avoid inducing an allergic or adverse drug reaction for which the patient is known to be at significant risk; (6) The team will consistently use methods known to minimize the risk for surgical site infection; (7) The team will prevent inadvertent retention of instrumentals or sponges in the surgical wounds; (8) The team will secure and accurately identify all surgical specimens; (9) The team will effectively communicate and exchange critical information for the safe conduct of the operation; and (10) Hospitals and public health systems will establish routine surveillance of surgical capacity, volume and results. 

Before data collection, face and content validity was performed with three nurses from a surgical center and two nurse professors with experience in the study theme, who were not included in the study. In addition, the judges performed a pre-test to ascertain the ease/difficulty in completing the instrument. As there were no disagreements, suggestions and difficulties in filling it out, no changes were required in the instrument.

The data were organized in a spreadsheet and the analysis was done with the use of Statistical Package for Social Sciences (SPSS) for Windows, version 19. The categorical variables were evaluated by means of absolute frequency and percentage. For the continuous variables, the position (mean, minimum and maximum) and dispersion (standard deviation) measurements were analyzed. In order to analyze the adherence of professionals to the objectives of the Initiative, a percentage of agreement equal or superior to 75% was set as adequate^7^.

The ethical recommendations were followed and the research was approved by the Research Ethics Committee through Certificate of Presentation for Ethical Appreciation (CAAE) no. 64255317.9.0000.0121. The Informed Consent Form was presented online to the participants before starting the data collection, in a clarification page about the research. The participant had to click at the option “I agree to participate in the survey” to confirm their agreement to the study terms and be directed to the next screen with the questionnaire.

## Results

A total of 248 responses were received, but the responses of 220 nurses were considered for the study sample. Based on the inclusion and exclusion criteria, we excluded 10 participants who reported having less than three months of experience in surgical center, 10 questionnaires due to double participation, and eight due to incomplete items. Table 1 shows the characterization of the socio-professional profile of the sample.

**Table 1:** Characterization of the socio-professional profile of the nurses participating in the study. Florianópolis, SC, Brazil, 2017

**Variable**	**n(%)**	**Mean**	**Standard deviation**	**Variation** **(min.-max.)**
**Age (years)**		**37.6**	**8.4**	**21-62**
**Sex**				
**Female**	**186(84.5)**			
**Male**	**34(15.5)**			
**Experience in surgical center (years)**		**7.6**	**7**	**0.25-37**
**Training**				
**Undergraduate course**	**31(14.2)**			
**Specialized in surgical center**	**75(34.2)**			
**Specialization in another area**	**62(28.3)**			
**Master**	**39(17.8)**			
**Phd**	**12(5.5)**			
**Region**				
**North**	**12(5.5)**			
**Northeast**	**29(13.2)**			
**Central-West and Federal District**	**9(4.1)**			
**Southeast**	**86(39.1)**			
**South**	**84(38.2)**			
**Type of institution**				
**Private**	**86(39.1)**			
**Public**	**76(34.5)**			
**Philanthropic**	**34(15.5)**			
**Public-Private**	**24(10.9)**			
**Area of performance**				
Only CC*	**16(7.3)**			
**Only PAR** ^**†**^	**6(2.7)**			
SC* and PAR ^**†**^	**60(27.3)**			
SC*, PAR ^**†**^ **and MSC** ^**‡**^	**86(39.1)**			
SC* and another unit	**52(23.6)**			
**Surgical rooms under their responsibility**		**6**	**3.9**	**0-28**
**Average volume of surgeries per month**		**468.79**	**482.9**	**6-3000**
**Type of professional performance**				
**Care nurse**	**117(53.2)**			
**Manager nurse**	**103(46.8)**			
**Weekly workload (in hours)**		**36.6**	**9.1**	**8-60**

*Surgical Center; †Post-Anesthesia Recovery; ‡Material and Sterilization Center.

Table 2 shows the distribution of responses regarding adherence to the 10 objectives of Safe Surgery Saves Lives Initiative. The highest level of agreement was evidenced in objective 1, with 144 (65.5%) respondents fully and 52 (23 .6%) partially agreeing with it. The lowest percentage of total (n = 69, 31.4%) and partial (n = 81; 36.8%) agreement was recorded in objective 10. 

**Table 2:** Distribution of participants’ answers regarding adherence to the 10 objectives of Safe Surgery Saves Lives Initiative. Florianópolis, SC, Brazil, 2017

**Objective**	TD* **n(%)**	**PD** ^**†**^ **n(%)**	**N** ^**‡**^ **n(%)**	**PA** ^**§**^ **n(%)**	**TA** ^**||**^ **n(%)**
**1 - The team will operate on the correct patient the correct site**	**6(2.7)**	**10(4.5)**	**8(3.6)**	**52(23.6)**	**144(65.5)**
**2 - The team will use methods known to prevent harm from administration and anesthetics, while protecting the patient from pain**	**8(3.6)**	**12(5.5)**	**20(9.1)**	**64(29.1)**	**116(52.7)**
**3 - The team will recognize and effectively prepare for life-threatening loss of airway or respiratory function**	**6(2.7)**	**14(6.4)**	**17(7.7)**	**65(29.5)**	**118(53.6)**
**4 - The team will recognize and effectively prepare for risk of high blood loss**	**6(2.7)**	**15(6.8)**	**20(9.1)**	**66(30)**	**113(51.4)**
**5 - The team will avoid inducing an allergic or adverse drug reaction for which the patient is known to be at significant risk**	**5(2.3)**	**11(5.0)**	**14(6.4)**	**73(33.2)**	**117(53.2)**
**6 - The team will consistently use methods known to minimize the risk for surgical site infection**	**6(2.7)**	**17(7.7)**	**10(4.5)**	**74(33.6)**	**113(51.4)**
**7 - The team will prevent inadvertent retention of instrumentals or sponges in the surgical wounds**	**6(2.7)**	**14(6.4)**	**14(6.4)**	**65(29.5)**	**121(55)**
**8 - The team will secure and accurately identify all surgical specimens**	**5(2.3)**	**11(5)**	**18(8.2)**	**61(27.7)**	**125(56.8)**
**9 - The team will effectively communicate and exchange critical information for the safe conduct of the operation**	**5(2.3)**	**19(8.6)**	**17(7.7)**	**73(33.2)**	**106(48.2)**
**10 - Hospitals and public health systems will establish routine surveillance of surgical capacity, volume and results**	**13(5.9)**	**27(12.3)**	**30(13.6)**	**69(31.4)**	**81(36.8)**

*I Totally Disagree; †I Partially Disagree; ‡Neutral; §I Partially Agree; ||I Totally Agree.

## Discussion

This is the first study that analyzed adherence to the 10 objectives of the Safe Surgery Saves Lives Initiative in surgical centers from the perspective of nurses from different regions of Brazil. Thus, the results contribute both to the production of scientific knowledge about patient safety in a surgical center and to the practice of nurses and managers in this area of care. In addition, the research presents an overview of the socio-professional characterization of surgical center nurses in Brazil.

The sample of this study was composed mainly by female participants (n = 186; 84.5%), with a mean of 37.6 years of age. These results are in line with the sociodemographic profile of nurses in Brazil^9^. The majority of participants had a specialization in a surgical center (n = 75, 34.2%), worked in a private hospital (n = 86, 39.1%) and were care nurses (n = 117, 53.2%).

The number of surgical rooms under the responsibility of nurses ranged from zero to 28. Despite the importance of nurses in the management of care^10^, the response zero may indicate that some have not considered themselves directly responsible for the operating rooms and attribute such responsibility to other nurses or managers. 

Most of the answers came from the South and Southeast regions, which may be related to the greater number of hospitals and surgical centers in these places. In addition, there is a concentration of the number of nurses in large urban centers in Brazil^9^.

Most nurses worked in more than one unit in the institution, in addition to the Surgical Center (n = 138; 62.7%), mainly Post-Anesthesia Recovery and Material and Sterilization Center (n = 86; 39.1%). In this sense, working in more than one sector can negatively impact nurses’ control over the care environment^11^.

Regarding adherence to the 10 objectives of the Safe Surgery Saves Lives Initiative, with the exception of objective 10, the other objectives presented partial and total agreement rates above 75%. This result indicates an adequate level of adherence to nine of the 10 analyzed objectives^7^.

However, some serious adverse events related to surgical procedures should not occur. These are never events, such as surgery or other invasive procedure performed at the wrong site or wrong patient; wrong surgery or invasive procedure in a patient; unintentional retention of foreign body in a patient after surgery or invasive procedure; and intraoperative or immediately postoperative death of a patient, according to the classification of the American Society of Anesthesiology (ASA)^12^ ^-^ ^13^.

From this classification, it can be considered that objectives 1, 7 and 8 aim at the prevention of never events. Therefore, any option other than I Totally Agree (TA) indicated by the participants of this study regarding these objectives indicates a risk to patient safety. According to ANVISA, in Brazil, from 2014 to 2017, 19 intraoperative or immediately postoperative deaths occurred in ASA I patients, 66 reports of unintentional foreign body retention and 12 surgical procedures in the wrong site of the body^13^.

Similarly, a Brazilian study identified a 98% rate of adherence of the team in relation to the 10 goals proposed by the WHO through the checklist of safe surgery. However, many items were not adequately filled, evidencing failure in patient safety, especially in objectives 1, 4, 5, 7, 8 and 9^14^.

In the international context, Canadian researchers, analyzing 212 cases of patients submitted to emergency abdominal surgery, found that 51.9% had a non-fatal complication, 22.6% lost independence and 6.6% died at the hospital^15^. In the Netherlands, from the investigation of 67,630 surgical procedures, 2,563 incidents were identified, of which 34% (n = 877) resulted from non-compliance with institutional protocols by professionals^16^.

The following is a discussion of the results obtained by each of the 10 objectives of the Safe Surgery Saves Lives Initiative.

Objective 1 obtained the highest agreement rate (89.1%) in relation to the objectives analyzed. However, this result is worrying, as this objective refers to a never ending event. A study conducted in São Paulo, Brazil, showed that 55% of the nursing staff classified the absence of laterality as an adverse event^17^. A survey conducted with 502 Brazilian orthopedists showed that 40% reported not demarcating the surgery site and 40% said they had already performed surgery in the wrong place. Most of the participants reported never being trained to use the safe surgery protocol^18^. Although it is not a reality in Brazil, the demarcation of the surgical site by nurses can contribute to the safety of the surgery according to Swiss study results^19^.

Regarding Objectives 2, 3, 4 and 5, on average, 50% of participants reported fully agreeing that the team adheres to WHO recommendations. These four objectives refer to patient safety in the anesthetic procedure^1^, which may have contributed to similar levels of agreement between participants.

The pre-anesthetic consultation should be performed for patients submitted to elective procedures and enables the prevention of events related to anesthetic practices. It is important for risk assessment for difficult airways, identification of allergies or adverse reactions and prediction of possible blood loss during the surgical procedure^20^.

Difficult airway access generates complications that can result in death or brain damage, which are avoidable from the assessment of the airway before anesthetic induction^21^. In Brazil, there are technologies available for the prevention of difficult airways, including simple and economical alternatives that contribute to patient safety^22^.

The prevention of risks related to adverse events is a key point in the safety of the anesthetic act. A Brazilian study presented an overview of the occurrence of Perioperative Anaphylaxis (PEOA), which is a rare allergic reaction, but with a rapid and fatal onset. The incidence varies according to the country, being 1:1,250 to 1:13,000 surgeries. The main causes are muscle relaxants, latex and antibiotics^23^.

Approximately 15% to 40% of patients who undergo surgical procedures present anemia at the time of surgery^24^. From the pre-anesthetic consultation, it is possible to reverse the anemic condition of the patient in about 15 days. Preoperative anemia is directly related to blood transfusion in the surgical procedure, which is considered the main cause of postoperative morbidity and mortality^25^.

Another important aspect is the role of the anesthesiologist in the administration of anesthetic drugs. Although intravenous drug delivery protocols have not shown major changes in the last 60 years, there is still a high rate of errors related to medication in the anesthetic act^26^.

In Santa Catarina, Brazil, a study with 61 anesthetists showed that 91.8% had already committed more than one medication administration error. The main causes were distraction, fatigue or low severity of the patient^27^. In China, a study showed omission, incorrect dosage and medication substitutions as major causes of error in anesthetic medication^28^. Incorrect identification of ampules and syringes is also one of the main causes of medication error related to the anesthetic act^29^.

In view of the international scenario and the legislation in force in Brazil, the work of the nurse combined with the anesthesiologist is crucial in for the planning and organization of materials and equipment for the anesthetic procedure. In addition, in the United States and in some European countries, there is a legislation that defines the training and independent performance of the nurse, with care protocols that allow the elaboration of the anesthetic plan and autonomy for the execution of care during the surgical procedure^6^.

Objective 6 obtained 85% of agreement among the nurses. Surgical Site Infection (SSI) occurs in about 3% to 20% of surgical procedures, constituting the main cause of morbidity and mortality in modern medical care^30^. Most SSIs are preventable, especially from the conduction of prophylactic antibiotic^31^ ^-^ ^32^. In Brazil, the adherence rate to the use of prophylactic antibiotic therapy is 84%^33^. In Sweden, this rate is estimated at 92%^34^.

Objective 7 had an agreement rate of 84% and also referred to a never event. This result is worrying given the serious consequences of such an event on patients. Sponge counting is a low-cost practice that requires organization and a structured method, such as a printed form^1^ ^,^ ^35^. Retention of a sponge on a surgical wound generates a gossypiboma, which is a textile matrix wrapped by foreign body reaction. It occurs mainly in the intra-abdominal area and may present fistula, abscess or mass^36^.

The incidence of gossypiboma is underreported due to medical and legal implications. According to a literature review, its occurrence rate in abdominal operations is 1:1,000 to 1:1,500. The patient often becomes asymptomatic, which also contributes to underreporting^37^. A study carried out in Pakistan has shown that the occurrence of gossypiboma occurs mainly in emergency surgeries^38^.

In Objective 8, partial and total agreement rate was of 84.5%. This finding is in line with the results of a study with 31 nursing professionals from a surgical center in São Paulo, Brazil, of which 92.9% considered the inappropriate disposal of a surgical specimen a serious adverse event^15^. In Taiwan, of the 200,345 specimens collected at a medical center, 1023 were with misidentification^39^.

Objective 9 obtained 81.4% of agreement, the second lowest index among the evaluated objectives, indicating that communication problems are very frequent in a surgical center. A Dutch study associated 11% of adverse events occurred in the operating room with relationship problems and communication failures^14^. In Brazil, the lack of communication between the medical and nursing staff represents 32% of the causes of adverse events in a surgical center^15^.

Objective 10 presented the lowest level of agreement among the analyzed objectives. The sharing of information and the socialization of indicators encourages learning from error. In addition, ongoing notification and tracking strengthens the dissemination of the safety culture and engages team members in the development of best safety practices^33^. The monitoring of results in the surgical centers is important to enable managers and professionals for decision making in the surgical center^40^. 

Thus, the results of the present study contribute to evidence of the complexity of adherence to WHO recommendations in the Safe Surgery Saves Lives Initiative. In addition, the findings may help managers and health professionals in the development of strategies for patient safety in the surgical center, especially in relation to never events.

Regarding the limitations of this study, the interpretations of the results can be considered of restricted scope due to the cross-sectional cut of the research and to the adoption of a non-probabilistic convenience sample. It should be emphasized that this kind of sampling does not allow us to identify whether the selected people are really representative of the population. 

However, the characteristics of the sample of the present study can help in the estimation of sample numbers for future studies, since it was a large population, with a significant number from several Brazilian regions. In relation to internal validity, performing online data collection makes it difficult to control samples and populations, since the questionnaire can be completed by someone other than the professional. In addition, it is easier for the participant to refuse to participate or to leave the study in progress, as well as there is greater possibility of people interested in the subject to cross the composition of the sample.

## Conclusion

Appropriate adherence to nine of the 10 objectives of the Safe Surgery Saves Lives Initiative was found. The objective that presented unsatisfactory adherence concerns the adoption by hospitals and health systems of routine surveillance mechanisms on surgical capacity, volume and results. Thus, it is expected that this study may subsidize the discussion of strategies to increase patient safety in the surgical center, especially in relation to health surveillance and prevention of never events.
